# Transportation of nanomaterial Maxwell fluid flow with thermal slip under the effect of Soret–Dufour and second-order slips: nonlinear stretching

**DOI:** 10.1038/s41598-022-25600-9

**Published:** 2023-02-07

**Authors:** Nadeem Abbas, Wasfi Shatanawi, Taqi A. M. Shatnawi

**Affiliations:** 1 Department of Mathematics and Sciences, College of Humanities and Sciences, Prince Sultan University, Riyadh, 11586 Saudi Arabia; 2 Department of Medical Research, China Medical University Hospital, China Medical University, Taichung, 40402 Taiwan; 3 Department of Mathematics, Faculty of Science, The Hashemite University, P.O Box 330127, Zarqa, 13133 Jordan

**Keywords:** Mathematics and computing, Nanoscience and technology

## Abstract

In this study, impact of second order slip for Maxwell fluid at vertical exponential stretching sheet is deliberated. Dufour and Soret impact for vertical exponential stretching sheet under nonlinear radiation are deliberated. Thermal and concentration slips with viscous dissipation are taken into account under the Buongiorno’s model. Under the above assumptions, the differential model constructed using the boundary layer approximations using the governing equations. The similarities transformations are introduced which applied the differential model (partial differential equations) and developed the dimensionless differential equations (ordinary differential equations). The dimensionless differential equations are cracked by numerical scheme. The impact of physical parameters are presented by tables and graphs. The curves of fluid velocity enhanced due to increasing the values of velocity slip. Velocity slip is a fluid-boundary interaction in physics. If the velocity slip increased, the fluid velocity profile would eventually become increasing. Temperature curves declined by improving values of ${K}_{1}$. The thermal thickness reduced when improved the values of ${K}_{1}$.

## Introduction

The non-Newtonian fluid have been achieved key role in various fields of real life like as plastic polymers, drilling muds, optical fibers, metal spinning, cooling of metallic plates in cooling baths, hot rolling paper production and so on. According to past literature, a single model have no capability to predict the all features of the non-Newtonian fluids. Every non Newtonian fluids have its own properties and their significant role. Further, these models divided into three kinds namely: rate-, integral-and differential-type of fluids. The Maxwell fluid known as rate type fluid because this type of fluid predict the impact of relaxation time which cannot be predicted by other type of fluid. The Maxwell fluid model was predicted by early time known as Maxwell^1^. The preduction of Maxwell fluid model got more attention be several researchers due to lot of applications in fields engineering and science. The impact of upper convicted Maxwell fluid at moving plate presented by Sadeghy et al.^2^. The results of Deborah’s number and fraction factors have opposite behavior. The impact of nonlinear radiation and time dependent flow of Maxwell fluid discussed by Mukhopadhyay et al.^3^. The unsteady and Maxwell fluid parameters and skin friction have same behavior of increasing found in their investigations and also their results used in fabulous in the polymer industry fields because this phenomena exist due to heat transfer between the fluid and surface covering it. Nadeem et al.^4^ discussed the nanomaterial flow of Maxwell fluid at moving surface with MHD effect. Sharma et al.^5^ deliberated Maxwell fluid model with nanomaterial flow at stretching sheet. Nadeem et al.^6^ highlighted the impact of Maxwell micropolar fluid with stagnation region at Riga sheet. Kumar et al.^7^ studied the magnetic dipole using the Maxwell fluid at stretching sheet. Gowda et al.^8^ debated about Maxwell liquid model using the Casson nanomaterial fluid by stretching disks. The different fluid models have been studied using the non-Newtonian fluid and Newtonian fluid over stretching surface (see Refs.^9–15^).

In the field of the thermal system, conventional heat transfers of base fluid, such as engine oil, ethylene glycol, and water, have been crucial. These liquids' limited ability to transport heat results from their poor thermal performance. The thermal characteristics of conventional fluids can be developed by suspending metallic and non-metallic solid particles in them. Nanofluids are those fluids that have suspended base fluid and nanomaterial. Nanofluid was invented by Choi and Eastman^16^. This presentation was quite effective. High heat transfer efficiency can be attained when conventional liquids scatter these crystals, despite the fact that most solid particles have poorer thermal conductivities than typical heat transfer liquids. Chamkha^17^ studied the solar radiative effects for natural convection over a vertical sheet. Chamkha et al.^18^ studied the heat generation of nanofluid flow at porous surface. Magyari and Chamkha^19^ analyzed the impact of micropolar nanomaterial fluid flow under chemical reaction and heat generation. The heat transfer of microplar nanomaterial fluid at Riga sheet highlighted by Nadeem at el.^20^. The modified nanomaterial fluid under thermal slip is deliberated by Nadeem et al.^21^. The nonlinear stretching sheet for nanomaterial fluid by Alblawi et al.^22^. The numerical results of nanomaterial fluid is studied by Awan et al.^23^. Awan et al.^24^ investigated the Jeffrey nanofluid at a stretching sheet. Awan et al.^25^ investigated the impact of non-Newtonian fluid flow over oscillatory stretching sheet. Nadeem et al.^26^ premeditated the influence of nanomaterial fluid flow at curved surface. Nadeem et al.^27^ premeditated the flow of non Newtonian in the presence of stagnation point region. Abbas et al.^28^ discussed the phase flow model of nanofluid at vertical wedge. Kumar et al.^29^ explored the results of nanomaterial fluid under MHD effect at stretching sheet. Punith et al.^30^ highlighted effects of Dufour and Soret with convective effects at stretching sheet. Kavya et al.^31^ explored the influence of MHD nanomaterial fluid flow at shrinking cylinder. Upadhya et al.^5^ explored the phase flow of casson micropolar fluid flow under entropy generation. Sharma et al.^32^ studied the Maxwell fluid flow at stretching sheet. Recently, numerous investigators have discussed the flow behavior under the different assumptions see in Refs.^15,33–46^.

Investigation about magnetic field has been much attracted by several authors due to physically importance as well as engineering and chemistry namely: pumps, generators (MHD), bearings and so on. Chamkha^47^ studied the natural convection of hydro magneticin porous medium. Chamkha^48^ analyzed the MHD and Hall effects with free convection at porous surface. Takhar et al.^49^ highlighted the influence of MHD for time depend flow in semi-infinite plate. Chamkha^50^ highlighted the impact of MHD three dimensional flow of free convection at permeable sheet. Chamkha^51^ discussed the MHD thermal radiative impacts on permeable surface. Takhar et al.^52^ studied the influence of MHD rotating flow at moving surface under the Hall and free stream effects. Chamkha and Ben^53^ highlighted the influence of mixed convection MHD flow at porous surface with Soret and Dufour’s impacts. Modather et al.^54^ discussed the flow of MHD oscillatory flow with micropolar fluid at vertical permeable plate. VeeraKrishna et al.^55^ investigated the impacts of MHD Hall impacts for second grade fluid flow at porous surface. Krishna and Chamkha^56^ analyzed the flow of MHD rotating nanofluid in porous medium under the Hall effects. Kumar et al.^57^ initiated the Reiner–Philippoff fluid under the MHD and Cattaneo–Christov heat flux. Krishna et al.^58^ deliberated the time dependent MJHD flow through porous surface. Awan et al.^59^ highlighted the influence of MHD radiative flow of nanomaterial fluid under solar energy. Few investigators have been studied about MHD flow under several effects and fluid models see Refs.^60–66^.

**Figure 1:**
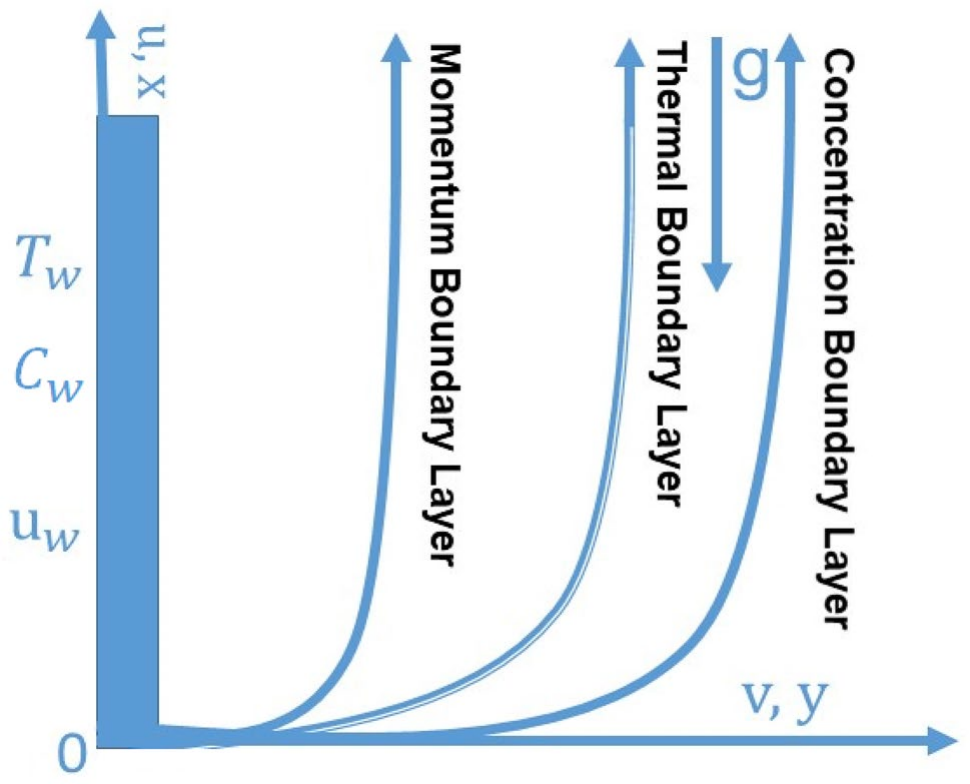
Flow pattern of Maxwell fluid.

Impact of second order slip for Maxwell fluid at vertical exponential stretching sheet is deliberated. Dufour and Soret impact for vertical exponential stretching sheet under nonlinear radiation are deliberated. Thermal and concentration slips with viscous dissipation are taken into account under the Buongiorno’s model. Under the above assumptions, the differential model constructed using the boundary layer approximations using the governing equations. The similarities transformations are introduced which applied the differential model (partial differential equations) and developed the dimensionless differential equations (ordinary differential equations). The dimensionless differential equations are cracked by numerical scheme. The impact of physical parameters are presented by tables and graphs. No one emphasized the Soret effects and second-grade slip at a vertically stretching sheet with Maxwell nanomaterial fluid flow. These findings could be applied to the industrial sector, which has shown to be more effective (Fig. 1).

## Flow analysis

The mathematical model of Maxwell fluid model is considered over vertical exponentially stretching sheet. The impact of second order slip with thermal and concentration slip are considered at the vertical exponential stretching sheet. The influence of Viscous dissipation and Soret effect with Buongiorno’s model to analysis the Brownian and thermophoresis. The differential system developed by using the boundary layer approximation after applying on governor models equations. ${u}_{w}$ is the wall stretching. ${T}_{\infty }$ is the ambient temperature. Impact of second order velocity, thermal and concentration slips under the bouncy forces are applied on the flow field. The succeeding equations are as bellow (see Refs.^15,43,44^):

The governing equations are presented as below:

$$divV=0,$$

$$\rho \frac{dV}{dt}=div\tau +\rho b,$$

The caushy stress tensor of the second grade fluid is defined as

$$\tau =-pI+\mu {B}_{1},$$

These tensors are defined as below:

$${B}_{1}+\lambda \left(\frac{dS}{dt}+SgradeV+{\left(gradeV\right)}^{T}S\right)=\mu {A}_{1}$$, and $${A}_{1}=gradeV+{(gradeV)}^{T}$$.

Here, material derivative ($\frac{d}{dt}$), pressure ($p$) and body forces ($b$). The material moduli must meet the given relationship of the Maxwell fluid model ab above. The velocity field and matrix transpose $V$ is presented as

$$V=\left[u\left(x, y, z\right), v\left(x, y, z\right), w\left(x, y, z\right)\right].$$

The energy equations for nanofluid is

$$\rho {c}_{p}\frac{dT}{dt}=-\nabla .q+{h}_{p}\nabla .{J}_{p},$$

Here, heat capacity ($${c}_{p}$$), specific entholpy ($${h}_{p}$$), temperature (T) of the nanoifluid, energy flux (q) and nanoparticles diffusion mass flux ($${J}_{p})$$ which presented as below:

$$\mathrm{q}= -\mathrm{k }\nabla T+{h}_{p}{J}_{p}, \,{J}_{p}= {\rho }_{p}{D}_{B}\nabla C- \frac{{\rho }_{p}{D}_{T}\nabla T}{{T}_{\infty }}.$$

The above equations is presentyed as energy equation of nanofluid. The concentration equation of nanofluid is

$$\mathrm{q}= -\mathrm{k }\nabla T+{h}_{p}{J}_{p}, \,{J}_{p}= {\rho }_{p}{D}_{B}\nabla C- \frac{{\rho }_{p}{D}_{T}\nabla T}{{T}_{\infty }}.$$

The terms are defined as thermal conductivity (k), nanoparticle mass density (${\rho }_{p}$), Brownian diffusion coefficient (${D}_{B}$), nanoparticle concentration (C) and thermophoretic diffusion coefficient (${D}_{T}$). Using the above equations

$$\frac{dT}{dt}=-k{\nabla }^{2}T+{\rho }_{p}{c}_{p}\left[{D}_{B}\nabla C\cdot \nabla T+{D}_{T}\frac{\nabla T\cdot \nabla T}{{T}_{\infty }}\right]+\frac{{D}_{T}{k}_{T}}{{\rho }_{f}{c}_{p}}\frac{{\partial }^{2}C}{\partial {y}^{2}}.$$

$$\frac{dC}{dt}={D}_{s}{\nabla }^{2}C+\frac{{D}_{T}{k}_{T}}{{T}_{\infty }}\frac{{\partial }^{2}T}{\partial {y}^{2}}.$$

The following assumptions are considered which are presented as below:

- Two-dimensional flow
- Viscous dissipation and second order slip
- Vertical exponential stretching sheet
- Buongiorno model
- Soret and Slip effects
- Maxwell fluid

The differential equation established and reduced by implementing the order of the approximation such as $O\left(u\right)=O\left(1\right), O\left(v\right)=O\left(\delta \right), O\left(x\right)=O\left(1\right), O\left(y\right)=O\left(\delta \right), O\left(T\right)=O\left(1\right), O\left(C\right)=O\left(1\right), O\left(\phi \right)=O\left(1\right)$. The reduced differential equations as following:

1 $$\frac{\partial u}{\partial x}+\frac{\partial v}{\partial y}=0,$$

2 $$u\frac{\partial u}{\partial x}+v\frac{\partial u}{\partial y}+{\delta }_{1}\left(\begin{array}{c}{u}^{2}\frac{{\partial }^{2}u}{\partial {x}^{2}}+{v}^{2}\frac{{\partial }^{2}u}{\partial {y}^{2}}+2uv\frac{{\partial }^{2}u}{\partial x\partial y}\end{array}\right) = \nu \left(\frac{{\partial }^{2}u}{\partial {y}^{2}}\right)-\frac{\sigma {{B}_{0}}^{2}}{{\rho }_{f}}u+g\left[{\beta }_{T}\left(T-{T}_{\infty }\right)+{\beta }_{C}\left(C-{C}_{\infty }\right)\right],$$

3 $$u\frac{\partial T}{\partial x}+v\frac{\partial T}{\partial y}={\alpha }_{f}\frac{{\partial }^{2}T}{\partial {y}^{2}}+\tau \left({D}_{B}\frac{\partial T}{\partial y}\frac{\partial \phi }{\partial y}+\frac{\tau {D}_{T}}{{T}_{\infty }}{\left(\frac{\partial T}{\partial y}\right)}^{2}\right)-\frac{\partial {q}_{r}}{\partial y}+\frac{\nu }{{\rho }_{f}{c}_{p}}{\left(\frac{\partial u}{\partial y}\right)}^{2}+\frac{{D}_{T}{k}_{T}}{{\rho }_{f}{c}_{p}}\frac{{\partial }^{2}C}{\partial {y}^{2}},$$

4 $$u\frac{\partial C}{\partial x}+v\frac{\partial C}{\partial y}={D}_{B}\left(\frac{{\partial }^{2}C}{\partial {y}^{2}}\right)+\frac{{D}_{T}{k}_{T}}{{T}_{\infty }}\frac{{\partial }^{2}T}{\partial {y}^{2}}.$$

The related boundary conditions

$$u={u}_{w}+A\frac{\partial u}{\partial y}+B\frac{{\partial }^{2}u}{\partial {y}^{2}}, v=0, {T}_{w}+\Delta \frac{\partial T}{\partial y}=T, C={C}_{w}+{\Delta }_{1}\frac{\partial C}{\partial y}, at \,y\to 0,$$

5 $$u\to 0,\, T\to {T}_{\infty }, \,C\to {C}_{\infty },\, at\, y\to \infty .$$

where, ${\rho }_{f}$, ${\rho }_{p}$, $\nu$, ${D}_{T}$, ${D}_{B}$, $A$, $B$, ${D}_{T}$ and $g$ presented as density of the fluid, density of nanoparticles, dynamic viscosity, thermophoretic, Brownian, first and second order velocity slip factor, Soret diffusivity and gravitational acceleration consistently. We defined resulting dimensionless variable (see Refs.^15,43,44^)

6 $$ \eta = \sqrt {\frac{{u_{0} }}{2\nu l}} yExp\left( \frac{x}{2l} \right),\, \theta \left( \eta \right) = \frac{{T - T_{\infty } }}{{T_{w} - T_{\infty } }}, \,u = u_{0} Exp\left( \frac{x}{l} \right)f^{^{\prime}} \left( \eta \right), \,v = - \sqrt {\frac{{\nu u_{0} }}{2l}} Exp\left( \frac{x}{2l} \right)\left( {f\left( \eta \right) + \eta f^{\prime } \left( \eta \right)} \right),\, \phi \left( \eta \right) = \frac{{C - C_{\infty } }}{{C_{w} - C_{\infty } }}. $$

The above system of differential equation reduced via the dimensionless variables as resulting

7 $$ f^{\prime \prime \prime } + ff^{\prime \prime } - 2f^{\prime } f^{\prime } + \left( {\gamma_{1} \theta + N_{c} \phi } \right) - Mf^{\prime } - \beta \left( {\begin{array}{*{20}c} {4f^{\prime 3} - \eta f^{\prime 2} f^{\prime \prime } - 6ff^{\prime } f^{\prime \prime } + f^{\prime \prime \prime } f^{2} } \\ \end{array} } \right) = 0, $$

8 $$ \left( {1 + \frac{4}{3}Rd} \right)\theta^{\prime \prime } + Pr\left( {\begin{array}{*{20}c} {f\theta^{\prime } - f^{\prime } \theta + Scf^{\prime \prime 2} + N_{B} \theta^{\prime } \phi^{\prime } + N_{T} \theta^{\prime 2} } \\ \end{array} } \right) + D_{f} \phi^{\prime \prime } = 0, $$

9 $$ \phi^{\prime \prime } + Sc\left( {f\phi^{\prime } - f^{\prime } \phi } \right) + ScS_{r} \theta^{\prime \prime } = 0, $$

And the relevant boundary conditions are at

10 $$ \begin{gathered}   f\left( \eta  \right) = 0,~~f^{'} \left( \eta  \right) = 1 + \lambda f^{{''}} \left( \eta  \right) + \lambda _{1} f^{{'''}} \left( \eta  \right), \hfill \\   K\left( {1 + \frac{4}{3}Rd} \right)\theta ^{\prime } \left( \eta  \right) + 1 = \theta \left( \eta  \right),~~\phi \left( \eta  \right) = 1 + K_{1} \phi ^{\prime } \left( \eta  \right),\,{\rm at} \eta- \hfill \\   f^{\prime}\left( \eta  \right) = 0,~\theta \left( \eta  \right) = 0,~\phi \left( \eta  \right) = 0,~~at~\eta  \to \infty ~~ \hfill \\  \end{gathered}$$

Here, derivative denoted as prime with respect to $\eta$. Magnetic field ($M=\frac{\sigma l{{B}_{0}}^{2}}{\rho {u}_{w}}$), Prandtl number ($Pr=\frac{\nu }{{\alpha }_{m}}$), Maxwell fluid parameter ($\beta =\frac{{\delta }_{1}{u}_{0}}{2l}$), Thermophoresis (${N}_{T}=\frac{\tau\Delta T{D}_{T}}{\nu {T}_{\infty }}$), Brownian motion (${N}_{B}= \frac{\tau\Delta C{D}_{B}}{\nu }$), Velocity slip ($\lambda =A\sqrt{\frac{{u}_{0}}{2\nu l}}$), Second order slip (${\lambda }_{1}=B\sqrt{\frac{{u}_{0}}{2\nu l}}$) and Bouncy force (${N}_{c}=\frac{{\beta }_{C}\left({T}_{w}-{T}_{\infty }\right)}{{\beta }_{T}\left({T}_{w}-{T}_{\infty }\right)}$).

## Physical quantities

The main physical expression of the fluid model is local Nusselt number and Sherwood number which are most vital role in this field. The expression of the following properties are presented as

11 $$N{u}_{x}=\frac{x{q}_{w}}{k(T)({T}_{w}-{T}_{\infty })} , S{h}_{x}=\frac{x{q}_{m}}{{D}_{S}({C}_{w}-{C}_{\infty })}.$$

In Eq. (14) ${q}_{w}$ and (Heat flux), ${q}_{m}$(Regular mass flux) are presented as

12 $${\left.{q}_{w}=-k\left(\frac{\partial T}{\partial y}\right)\right|}_{y=0}, {\left.{q}_{n}=-{D}_{S}\left(\frac{\partial C}{\partial y}\right)\right|}_{y=0}$$

In the dimensionless form,

13 $$ Nu_{x} \left( {Re_{x} } \right)^{{ - \frac{1}{2}}} = - \theta^{\prime}\left( 0 \right), Sh_{x} \left( {Re_{x} } \right)^{{ - \frac{1}{2}}} = - \phi^{\prime } \left( 0 \right). $$

The local Reynolds number is ${Re}_{x}={u}_{w}\sqrt{\frac{2l{u}_{o}}{\nu }}$.

## Solution procedure

Solving the dimensionless system of differential equations are reduced in first order using the bvp4c technique. The procedure of the following method is as (see Refs.^15,43,44^):

14 $$ \left( {\begin{array}{*{20}c} {\begin{array}{*{20}c} f \\ {f^{\prime } } \\ \end{array} } \\ {f^{\prime \prime } } \\ {f^{\prime \prime \prime } } \\ \theta \\ {\theta^{\prime } } \\ {\theta^{\prime \prime } } \\ \phi \\ {\phi^{\prime } } \\ {\phi^{\prime \prime } } \\ \end{array} } \right) = \left( {\begin{array}{*{20}c} {\begin{array}{*{20}c} {S\left( 1 \right)} \\ {S\left( 2 \right)} \\ \end{array} } \\ {S\left( 3 \right)} \\ {SS1} \\ {S\left( 4 \right)} \\ {S\left( 5 \right)} \\ {SS2} \\ {S\left( 6 \right)} \\ {S\left( 7 \right)} \\ {SS3} \\ \end{array} } \right); $$

15 $$SS1=\frac{-1}{1-\beta S\left(1\right)S\left(1\right)}\left(S\left(1\right)S\left(3\right)-2S\left(2\right)S\left(2\right)+\left({\gamma }_{1}S\left(4\right)+{N}_{c}S\left(6\right)\right)-MS\left(2\right)-\beta \left(\begin{array}{c}4S\left(2\right)S\left(2\right)S\left(2\right)-xS\left(2\right)S\left(2\right)S\left(3\right)-6S\left(1\right)S\left(2\right)S\left(3\right)\end{array}\right)\right);$$

16 $$SS2={\left(1+\frac{4}{3}Rd\right)}^{-1}\left(Pr\left(\begin{array}{c}S\left(1\right)S\left(5\right)-S\left(2\right)S\left(4\right)+ScS\left(3\right)S\left(3\right)+{N}_{B}S\left(7\right)S\left(5\right)+{N}_{T}S\left(5\right)S\left(5\right)\end{array}\right)+{D}_{f}SS3\right);$$

17 $$SS3=-\left(Sc\left(S(1)S(7)-S(2)S(6)\right)+Sc{S}_{r}SS2\right);$$

With boundary conditions are

18 $$S0\left(1\right); S0\left(2\right)-1-S0\left(3\right)-SS0\left(1\right); K\left(1+\frac{4}{3}Rd\right)S0\left(5\right)-1-S0\left(4\right); S0\left(6\right)-1-{K}_{1}S0\left(7\right);Sinf\left(2\right); Sinf\left(4\right);Sinf\left(6\right);$$

${R}_{1}\left(\overline{{u }_{1}}, \overline{{u }_{2}},\overline{{u }_{3}}\right), {R}_{2}\left(\overline{{u }_{1}}, \overline{{u }_{2}},\overline{{u }_{3}}\right)$, ${R}_{3}\left(\overline{{u }_{1}}, \overline{{u }_{2}},\overline{{u }_{3}}\right)$ are the residual of present model and non linear differential equations are solved by RK-4^th^ order. If the solution is converge when tolerance error i.e., than ${10}^{-6}$. The boundary residuals are exhibited as:

$${R}_{1}\left(\overline{{u }_{1}}, \overline{{u }_{2}},\overline{{u }_{3}}, \overline{{u }_{4}}\right) =\left|{\mathrm{S}}_{2}\left(\infty \right)-\widehat{{\mathrm{S}}_{2}}\left(\infty \right)\right|;$$

$${R}_{2}\left(\overline{{u }_{1}}, \overline{{u }_{2}},\overline{{u }_{3}}, \overline{{u }_{4}}\right) =\left|{\mathrm{S}}_{4}\left(\infty \right)-\widehat{{\mathrm{S}}_{4}}\left(\infty \right)\right|;$$

$${R}_{3}\left(\overline{{u }_{1}}, \overline{{u }_{2}},\overline{{u }_{3}}, \overline{{u }_{4}}\right) =\left|{\mathrm{S}}_{6}\left(\infty \right)-\widehat{{\mathrm{S}}_{6}}\left(\infty \right)\right|;$$

Hence, $\widehat{{S}_{2}}\left(\infty \right), \widehat{{S}_{4}}\left(\infty \right), \widehat{{S}_{6}}\left(\infty \right)$ are computed boundary values.

## Results and discussion

**Figure 2:**
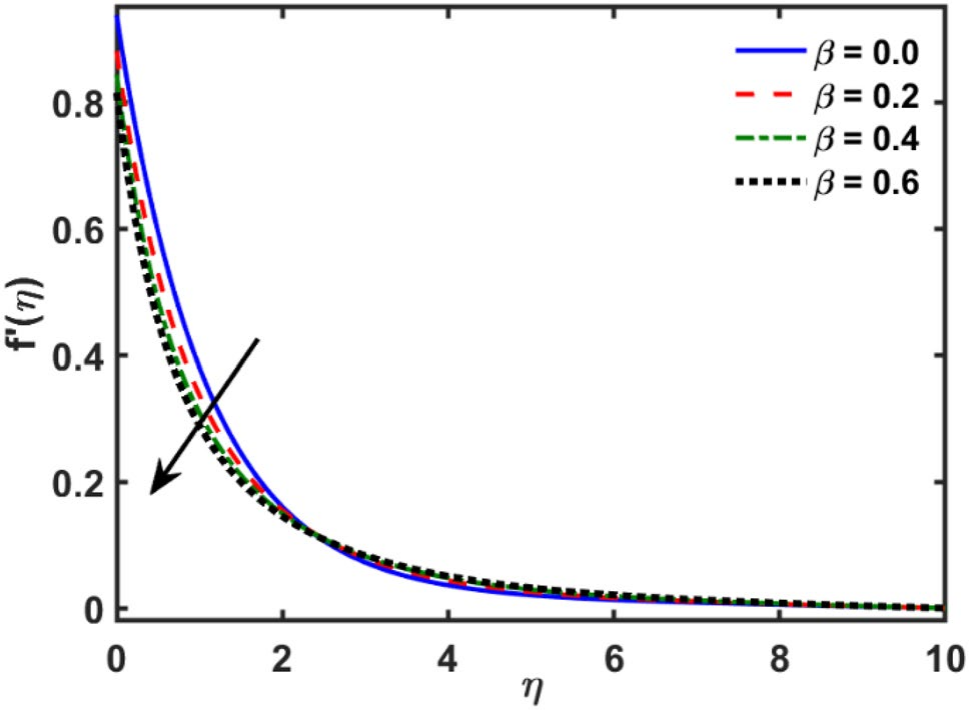
Variation of $\beta$ on $f^{\prime } \left( \eta \right)$.

**Figure 3:**
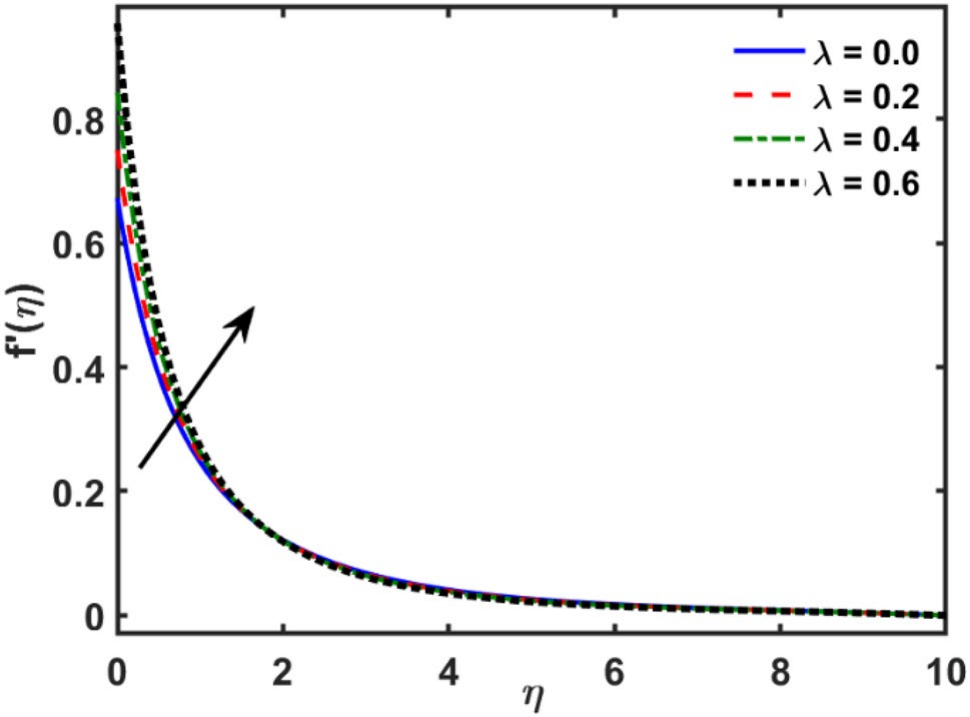
Variation of $\lambda$ on $f^{\prime } \left( \eta \right)$.

**Figure 4:**
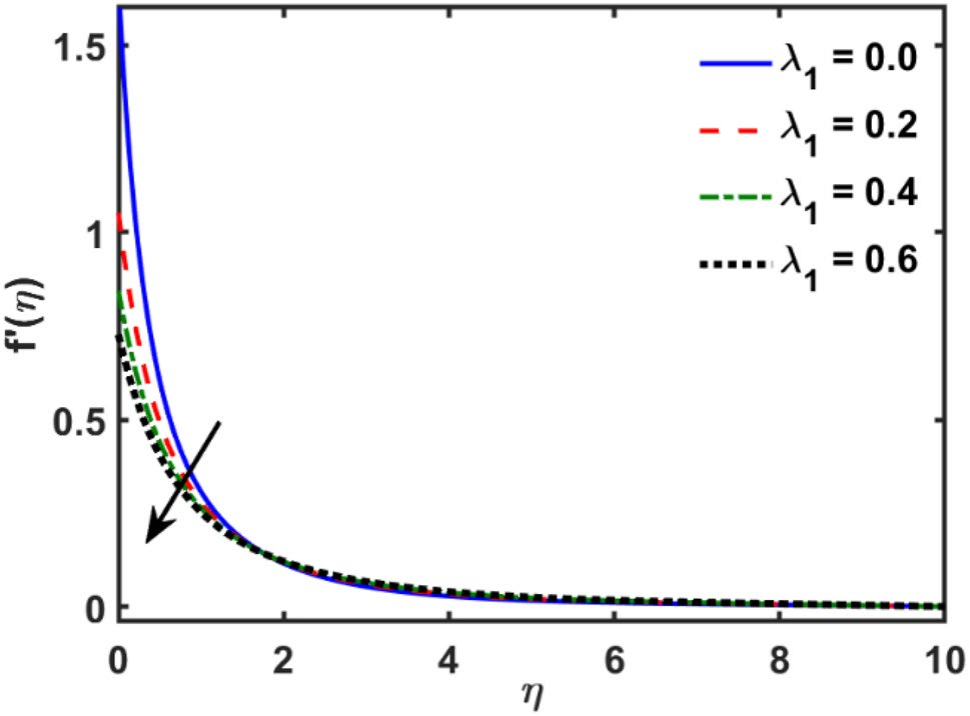
Variation of $\lambda_{1}$ on $f^{\prime } \left( \eta \right)$.

**Figure 5:**
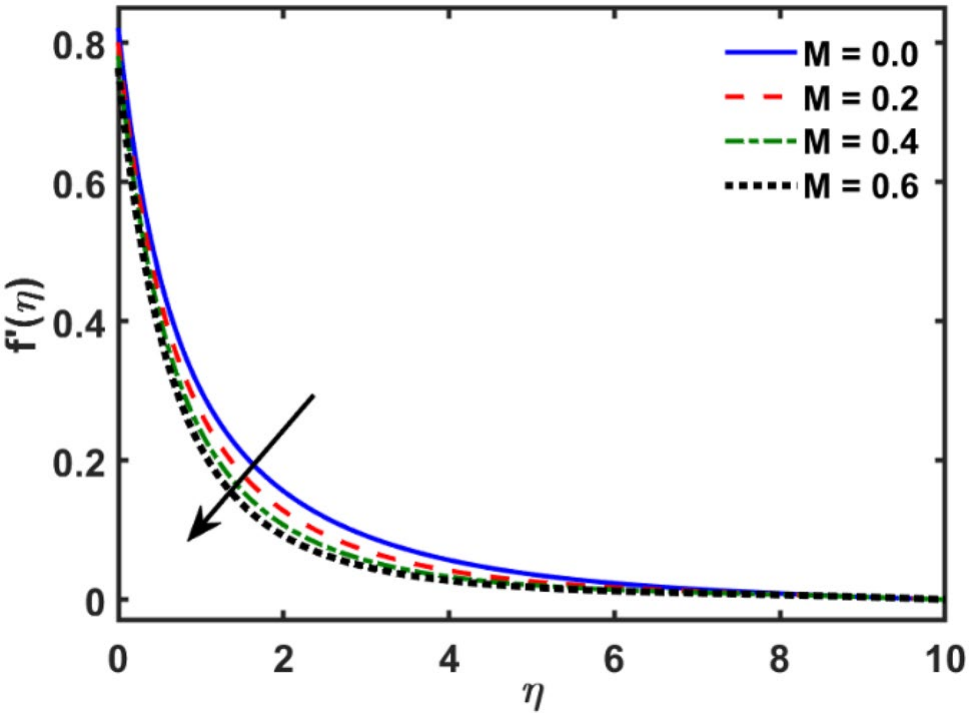
Variation of $M$ on $f^{\prime } \left( \eta \right)$.

**Figure 6:**
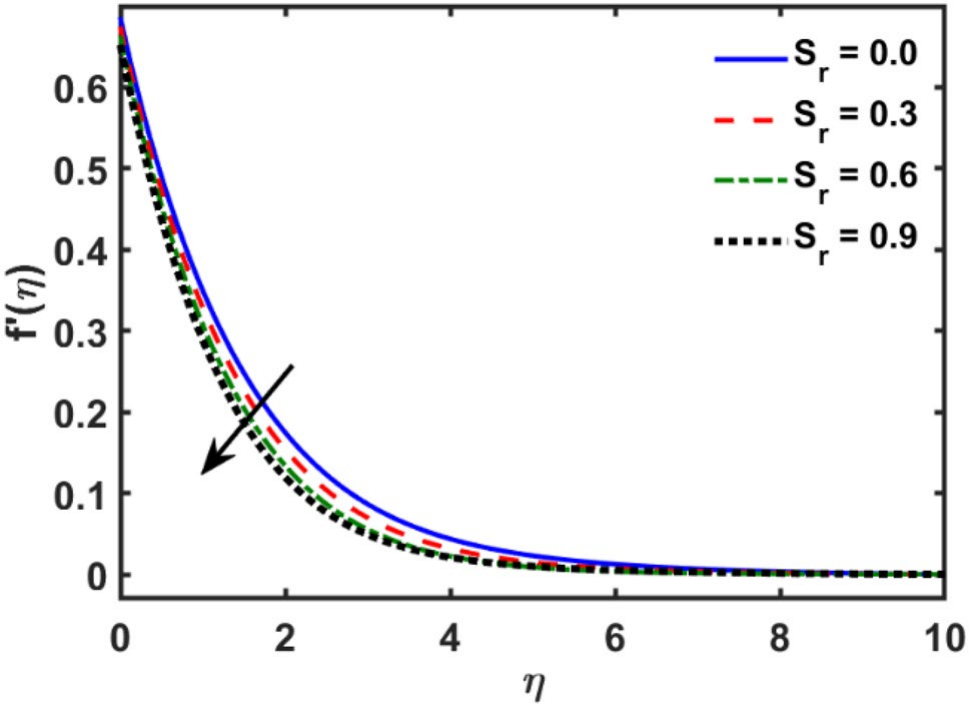
Variation of $S_{r}$ on $f^{\prime } \left( \eta \right)$.

**Figure 7:**
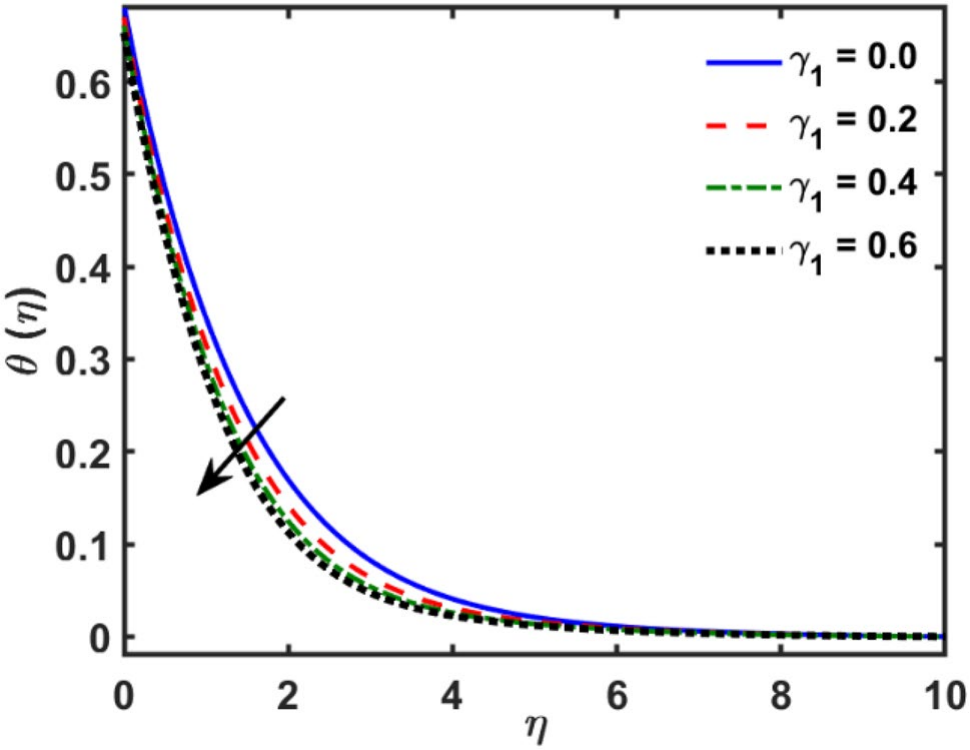
Variation of $\gamma_{1}$ on $\theta \left( \eta \right)$.

**Figure 8:**
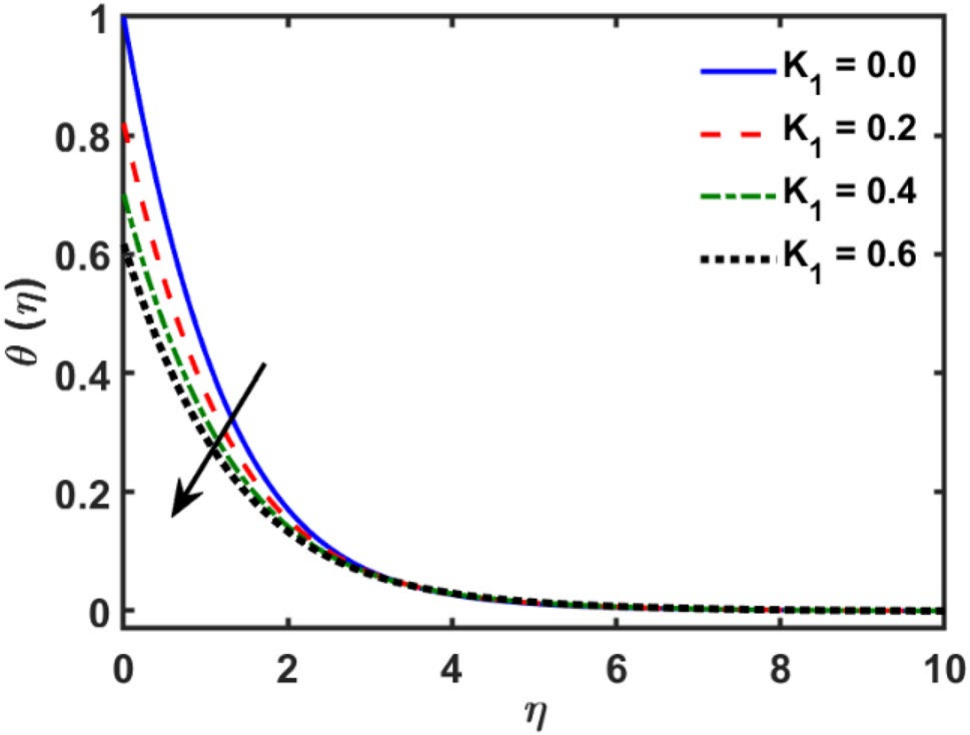
Variation of $K_{1}$ on $\theta \left( \eta \right)$.

**Figure 9:**
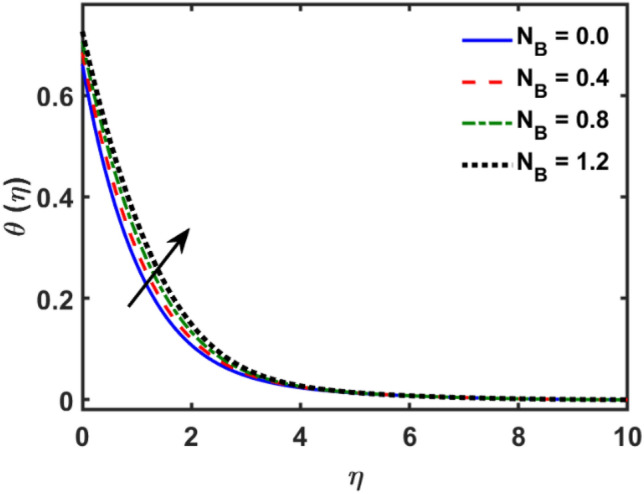
Variation of $N_{B}$ on $\theta \left( \eta \right)$.

**Figure 10:**
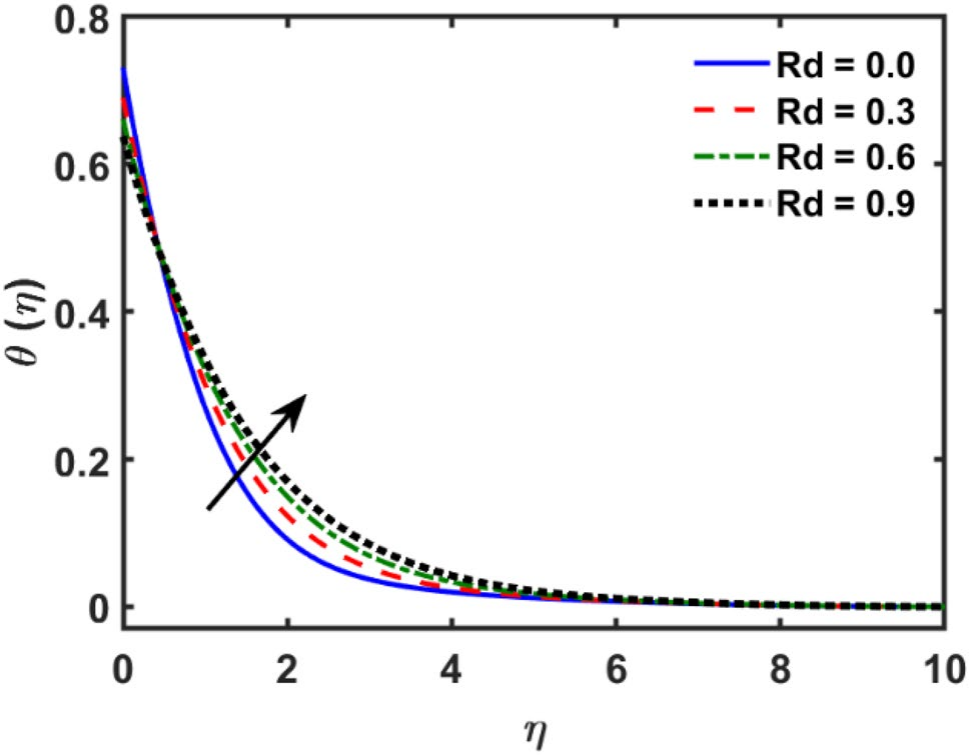
Variation of $Rd$ on $\theta \left( \eta \right)$.

**Figure 11:**
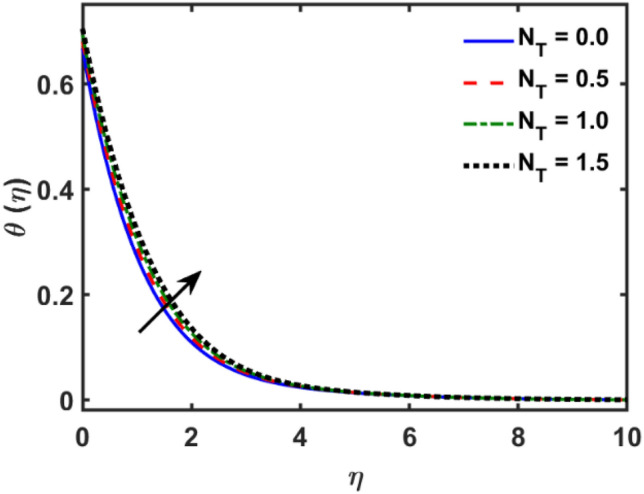
Variation of $N_{T}$ on $\theta \left( \eta \right)$.

**Figure 12:**
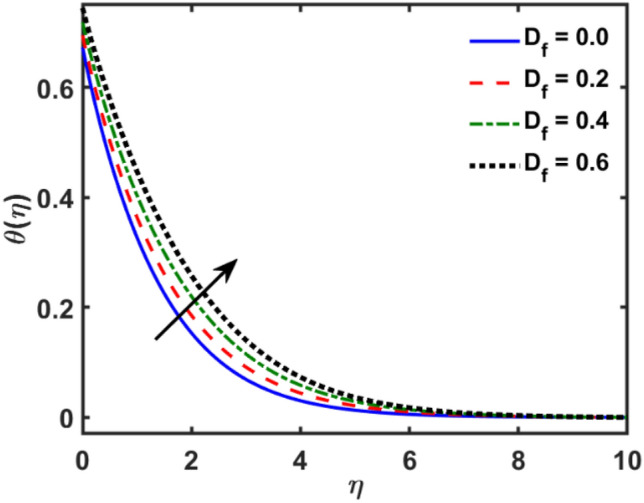
Variation of $D_{f}$ on $\theta \left( \eta \right)$.

**Figure 13:**
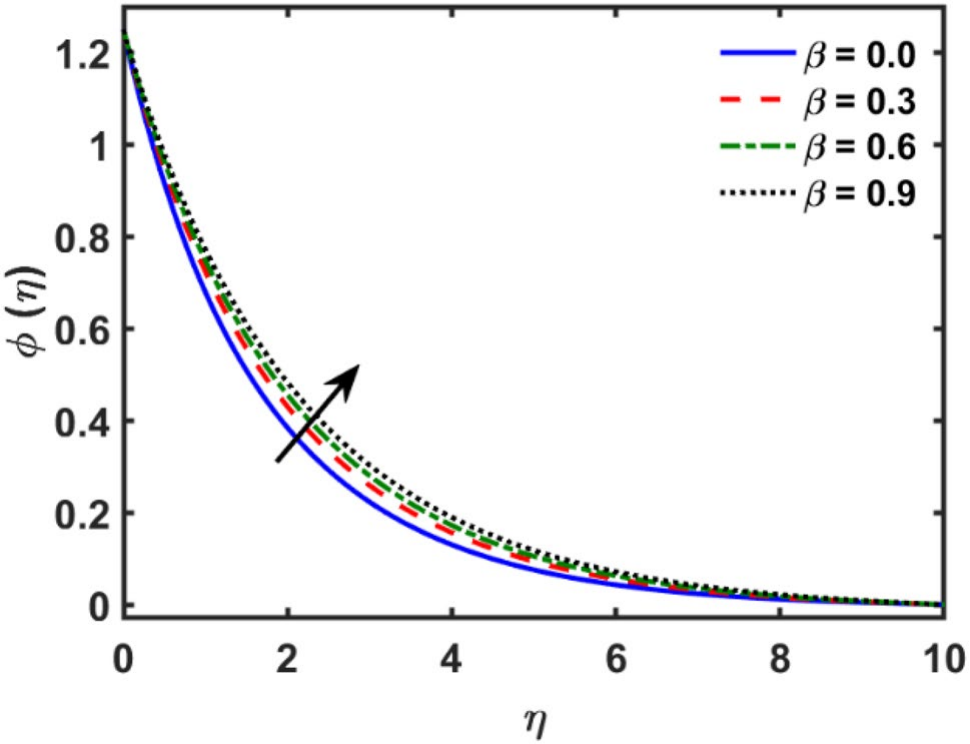
Variation of $\beta$ on $\phi \left( \eta \right)$.

**Figure 14:**
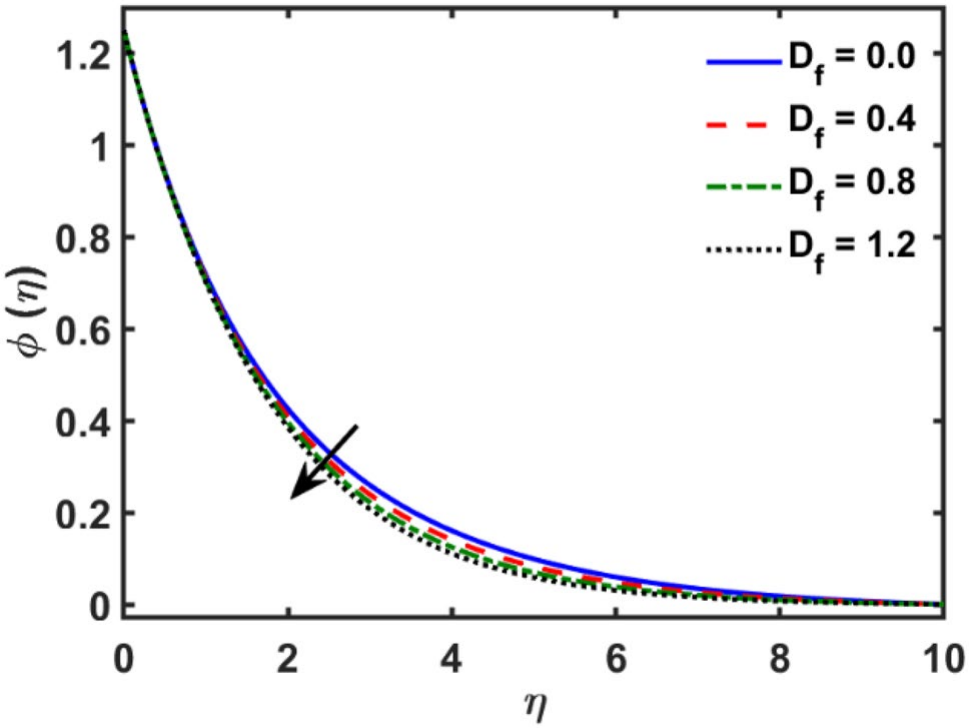
Variation of $D_{f}$ on $\phi \left( \eta \right)$.

**Figure 15:**
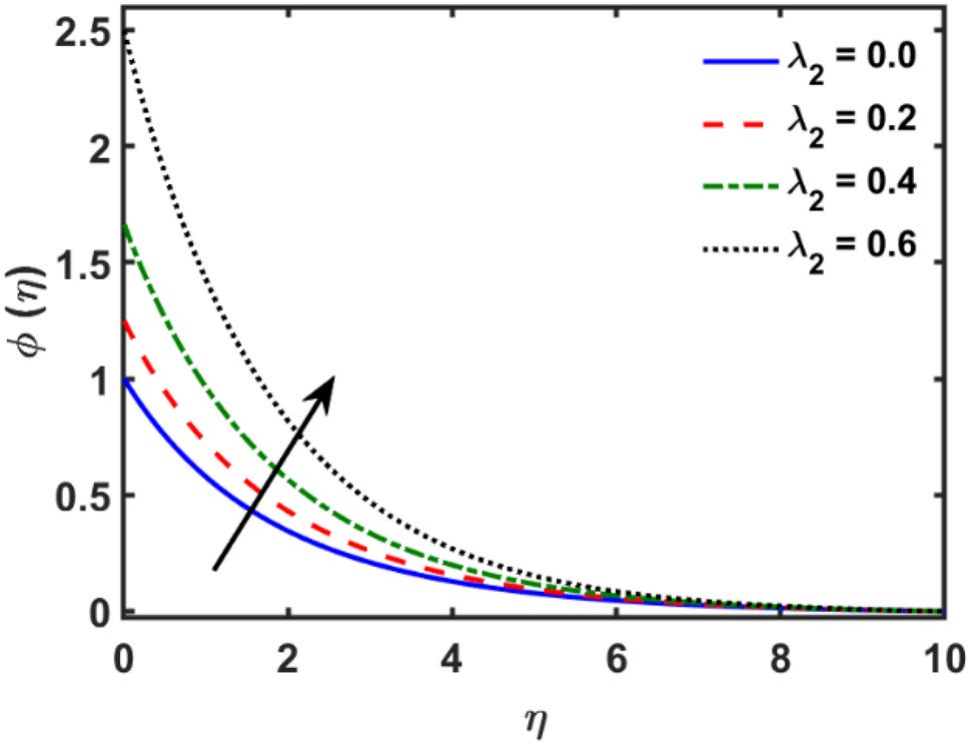
Variation of $\lambda_{2}$ on $\phi \left( \eta \right)$.

**Figure 16:**
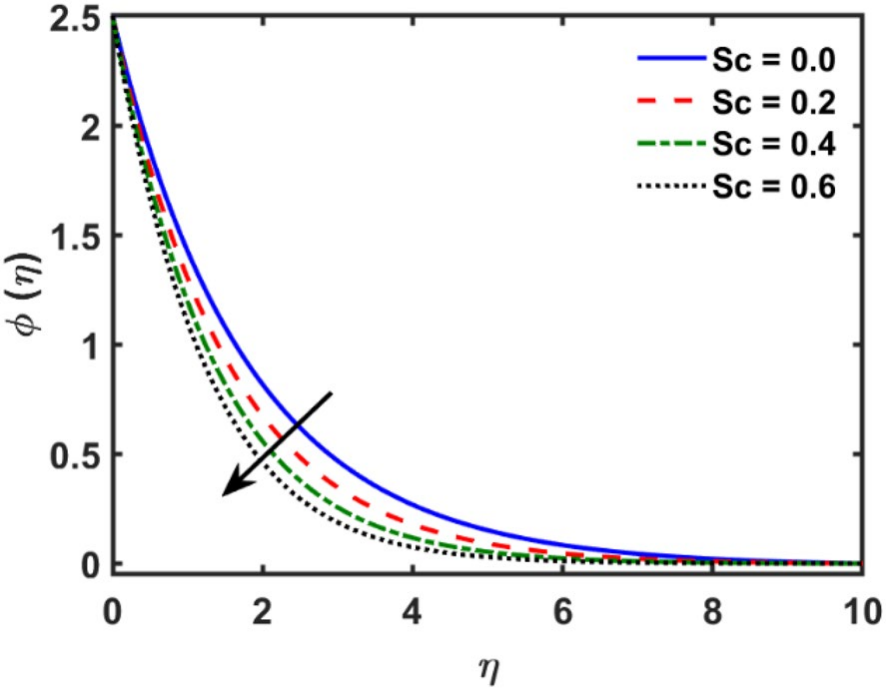
Variation of $Sc$ on $\phi \left( \eta \right)$.

**Figure 17:**
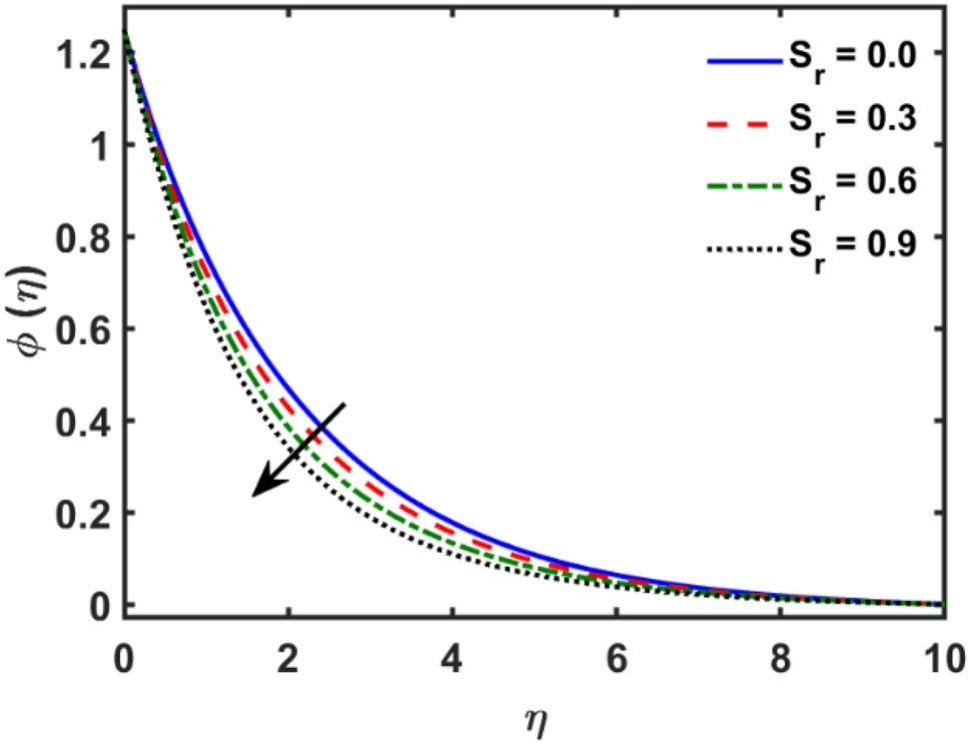
Variation of $S_{r}$ on $\phi \left( \eta \right)$.

Figures 2, 3, 4, 5, 6, 7, 8, 9, 10, 11, 12, 13, 14, 15, 16 and 17 depicted the physical influence of parameters on the velocity, temperature and concentration functions. Figures 2, 3, 4, 5 and 6 presented the impact of $\beta$, $\lambda$. ${\lambda }_{1}, M$ and ${S}_{r}$ on the velocity function. Figure 2 reveals the variation of $\beta$ and velocity function. The fluid velocity function revealed the curves declining due to boosting values of $\beta$. The variation of $\lambda$ and fluid velocity function presented in Fig. 3. The curves of fluid velocity enhanced due to increasing the values of $\lambda$. Velocity slip is a fluid-boundary interaction in physics. If the velocity slip increased, the fluid velocity profile would eventually become increasing. Second order slip ${\lambda }_{1}$ influence on fluid velocity presented in Fig. 4. Fluid velocity curves revealed declining behavior due to improving the ${\lambda }_{1}$. The impact of $M$ on fluid velocity presented in Fig. 5. The velocity of fluid declined by boosting values of $M$. In fact, increasing magnetic field value increases the external magnetic field. This increase in the external magnetic field causes a wall parallel Lorentz force, which slows the expansion of the momentum boundary layer. When looking at the figure closely, it can be seen that the velocity suddenly declined towards the plate as the magnetic field increases. The variation of ${S}_{r}$ and fluid velocity presented in Fig. 6. Increasing of ${S}_{r}$ which lessened the curves of fluid velocity. Due to crass diffusion impacts, the ${S}_{r}$ (thermo diffusion) increasing which resist to fluid velocity as well as fluid velocity declined. Figures 7, 8, 9, 10, 11 and 12 depicted the impact of ${\gamma }_{1}$, ${K}_{1}$, ${N}_{B}$, $Rd$, ${N}_{T}$ and ${D}_{f}$ on the fluid temperature. Figure 7 depicted the variation of ${\gamma }_{1}$ and temperature function. Temperature curves declined by improving values of ${\gamma }_{1}$. Figure 8 depicted the variation of ${K}_{1}$ and temperature function. Temperature curves declined by improving values of ${K}_{1}$. The thermal thickness reduced when improved the values of ${K}_{1}$. Figure 9 depicted the ${N}_{B}$ on temperature function. Temperature function curved boosted due to boosting values of ${N}_{B}$. Physically, the Brownian motion developed the kinetic energy as well as Brownian motion increased which increased the kinetic energy ultimately temperature of fluid increased. Impact of $Rd$ o fluid temperature depicted in Fig. 10. Values of $Rd$ and fluid temperature found to be same behavior of increasing found. Physically, radiation increasing means energy increased as well as temperature of fluid increased. Figure 11 revealed the impact of ${N}_{T}$ on temperature function. Temperature function increased due to improving values of ${N}_{T}$. As the values of ${N}_{T}$ increased which enhanced the temperature due to ${N}_{T}$ has high gradient temperature. The variation of ${D}_{f}$ and temperature function depicted in Fig. 12. The values of ${D}_{f}$ and fluid temperature found to be same behavior of increasing. Thermal thickness increased as well as ${D}_{f}$ (diffusion-thermo) increased. The impact of $\beta$, ${D}_{f}$, ${\lambda }_{2}$, $Sc$ and ${S}_{r}$ on concentration function which presented in Figs. 13, 14, 15, 16 and 17. Impact of $\beta$ on fluid concentration function presented in Fig. 13. Curves of fluid concentration declined due to boosting values of $\beta$. Variation of ${D}_{f}$ on the fluid concentration presented in Fig. 14. The ${D}_{f}$ parameter increased which declined the curves of fluid concentration. Physically, ${D}_{f}$ (diffusion-thermo) values enhanced which reduced the concentration function. Influence of ${\lambda }_{2}$ on fluid concentration presented in Fig. 15. It is seen that curves of fluid concentration increased due to improving values of ${\lambda }_{2}.$ The variation of $Sc$ and fluid concentration presented in Fig. 16. The values of fluid concentration declined due to improving values of $Sc$. Influence of ${S}_{r}$ on the fluid concentration presented in Fig. 17. Fluid concentration curves declined due to boosting values of ${S}_{r}$.

Table 1 presented the influence of $\beta , M,$ ${\gamma }_{1}$, $\lambda , {\lambda }_{1}$ and ${N}_{c}$ on magnitude of $f^{\prime \prime } \left( 0 \right)$ while the other values fixed with ${D}_{f}$, ${S}_{r}$, $Rd$, ${N}_{B}$, ${N}_{T}$, $Sc$, $K$, ${K}_{1}$ and $Pr$. The influence of $\beta$ on the magnitude of $f^{\prime \prime } \left( 0 \right)$ presented in Table 1 when the values of $=0.3,$ ${\gamma }_{1}=0.3$, $\lambda =0.4$, ${\lambda }_{1}=0.4$ and ${N}_{c}=0.2$ are fixed. The magnitude of magnitude of $f^{\prime \prime } \left( 0 \right)$ increased for different values of $\beta$. The influence of $M$ on the magnitude of $f^{\prime \prime } \left( 0 \right)$ presented in Table 1 when the values of $=0.3,$ ${\gamma }_{1}=0.3$, $\lambda =0.4$, ${\lambda }_{1}=0.4$ and ${N}_{c}=0.2$ are fixed. The magnitude of $f^{\prime \prime } \left( 0 \right)$ increased for different values of $M$. Variation of ${\gamma }_{1}$ and magnitude of $f^{\prime \prime } \left( 0 \right)$ presented in Table 1 and other parameter are fixed such as $=0.3, M=0.3,$ $\lambda =0.4$, ${\lambda }_{1}=0.4$ and ${N}_{c}=0.2$. The magnitude of $f^{\prime \prime } \left( 0 \right)$ revealed the decline behavior for increment in ${\gamma }_{1}$. Impact of ${\lambda }_{1}$ on the magnitude of $f^{\prime \prime } \left( 0 \right)$ with fix values of $=0.3, M=0.3,$ ${\gamma }_{1}=0.3$, $\lambda =0.4$ and ${N}_{c}=0.2$ presented in Table 1. The magnitude of $f^{\prime \prime } \left( 0 \right)$ revealed the declining behavior for increment in ${\lambda }_{1}$. Impact of $\lambda$ on the magnitude of $f^{\prime \prime } \left( 0 \right)$ with fix values of $=0.3, M=0.3,$ ${\gamma }_{1}=0.3$, ${\lambda }_{1}.=0.4$ and ${N}_{c}=0.2$ presented in Table 1. The magnitude of $f^{\prime \prime } \left( 0 \right)$ revealed the increasing behavior for increment in $\lambda$. Impact of $\lambda$ on the magnitude of $f^{\prime \prime } \left( 0 \right)$ with fix values of $=0.3, M=0.3,$ ${\gamma }_{1}=0.3$, ${\lambda }_{1}.=0.4$ and $\lambda =0.4$ presented in Table 1. The magnitude of $f^{\prime \prime } \left( 0 \right)$ revealed the deteriorating behavior for increment in ${N}_{c}$.

**Table 1:** Numerical results of $f^{\prime \prime } \left( 0 \right)$ for different values of physical parameters.

$\beta$	$M$	${\gamma }_{1}$	${\lambda }_{1}$	$\lambda$	${N}_{c}$	$f^{\prime \prime}(0)$
0.1	0.3	0.3	0.4	0.4	0.2	-0.9442
0.2	-	-	-	-	-	-0.9972
0.3	-	-	-	-	-	-1.0397
0.4	-	-	-	-	-	-1.0756
0.3	0.0	-	-	-	-	-0.9976
-	0.3	-	-	-	-	-1.0397
-	0.6	-	-	-	-	-1.0791
-	0.9	-	-	-	-	-1.1158
-	0.3	0.0	-	-	-	-1.1847
-	-	0.3	-	-	-	-1.0397
-	-	0.6	-	-	-	-0.9304
-	-	0.9	-	-	-	-0.8360
-	-	0.3	0.0	-	-	-3.1739
-	-	-	0.2	-	-	-1.5266
-	-	-	0.4	-	-	-1.0397
-	-	-	0.6	-	-	-0.7952
-	-	-	0.4	0.0	-	-0.5714
-	-	-	-	0.2	-	-0.7746
-	-	-	-	0.4	-	-1.0397
-	-	-	-	0.6	-	-1.3916
-	-	-	-	0.4	0.0	-1.0843
-	-	-	-	-	0.2	-1.0397
-	-	-	-	-	0.4	-0.9988
-	-	-	-	-	0.6	-0.9603

Table 2 presented the impact of ${D}_{f}$, ${S}_{r}$, $Rd$, ${N}_{B}$, ${N}_{T}$, $Sc$, $K$, ${K}_{1}$ and $Pr$ on the magnitude of $\theta^{\prime } \left( 0 \right)$ and $\phi^{\prime } \left( 0 \right)$ with fix values of $\beta =0.3, M=0.3,$ ${\gamma }_{1}=0.3$, ${\lambda }_{2}=0.4$, ${\lambda }_{1}=0.4$ and ${N}_{c}=0.2$. The variation of ${D}_{f}$ and the magnitude of $\theta^{\prime } \left( 0 \right)$ and $\phi^{\prime } \left( 0 \right)$ presented in Table 2. The magnitude of $\theta^{\prime } \left( 0 \right)$ declined but magnitude of $\phi^{\prime } \left( 0 \right)$ increased by improving values of ${D}_{f}$ with fix values of ${S}_{r}=0.3$, $Rd=0.3$, ${N}_{B}=0.2$, ${N}_{T}=0.4$, $Sc=0.7$, $K=0.3$, ${K}_{1}=0.6$ and $Pr=1.5$. The variation of ${S}_{r}$ and the magnitude of $\theta^{\prime } \left( 0 \right)$ and $\phi^{\prime } \left( 0 \right)$ presented in Table 2. The magnitude of $\theta^{\prime } \left( 0 \right)$ and $\phi^{\prime } \left( 0 \right)$ increased by improving values of ${S}_{r}$ with fix values of ${D}_{f}=0.2$, $Rd=0.3$, ${N}_{B}=0.2$, ${N}_{T}=0.4$, $Sc=0.7$, $K=0.3$, ${K}_{1}=0.6$ and $Pr=1.5$. The variation of $Rd$ and the magnitude of $\theta^{\prime } \left( 0 \right)$ and $\phi^{\prime } \left( 0 \right)$ presented in Table 2. The magnitude of $\theta^{\prime } \left( 0 \right)$ reduced and magnitude of $\phi^{\prime } \left( 0 \right)$ increased by improving values of $R$ with fix values of $D_{f} = 0.2$, $S_{r} = 0.3$, $N_{B} = 0.2$, $N_{T} = 0.4$, $Sc = 0.7$, $K = 0.3$, $K_{1} = 0.6$ and $Pr = 1.5$. The variation of $N_{B}$ and the magnitude of $\theta^{\prime } \left( 0 \right)$ and $\phi^{\prime } \left( 0 \right)$ presented in Table 2. The magnitude of $\theta^{\prime } \left( 0 \right)$ reduced and magnitude of $\phi^{\prime } \left( 0 \right)$ increased by improving values of $N_{B}$ with fix values of $D_{f} = 0.2$, $S_{r} = 0.3$, $Rd = 0.3$, $N_{T} = 0.4$, $Sc = 0.7$, $K = 0.3$, $K_{1} = 0.6$ and $Pr = 1.5$. The variation of $N_{T}$ and the magnitude of $\theta^{\prime } \left( 0 \right)$ and $\phi^{\prime } \left( 0 \right)$ presented in Table 2. The magnitude of $\theta^{\prime } \left( 0 \right)$ reduced and magnitude of $\phi^{\prime } \left( 0 \right)$ increased by improving values of $N_{T}$ with fix values of $D_{f} = 0.2$, $S_{r} = 0.3$, $Rd = 0.3$, $N_{b} = 0.2$, $Sc = 0.7$, $K = 0.3$, $K_{1} = 0.6$ and $Pr = 1.5$. The variation of $Sc$ and the magnitude of $\theta^{\prime } \left( 0 \right)$ and $\phi^{\prime } \left( 0 \right)$ presented in Table 2. The magnitude of $\theta^{\prime } \left( 0 \right)$ reduced and magnitude of $\phi^{\prime } \left( 0 \right)$ increased by improving values of $Sc$ with fix values of $D_{f} = 0.2$, $S_{r} = 0.3$, $Rd = 0.3$, $N_{b} = 0.2$, $N_{T} = 0.4$, $K = 0.3$, $K_{1} = 0.6$ and $Pr = 1.5$. The variation of $K$ and the magnitude of $\theta^{\prime } \left( 0 \right)$ and $\phi ^{\prime}\left( 0 \right)$ presented in Table 2. The magnitude of $\theta^{\prime } \left( 0 \right)$ and $\phi^{\prime } \left( 0 \right)$ reduced by improving values of $K$ with fix values of $D_{f} = 0.2$, $S_{r} = 0.3$, $N_{B} = 0.2$, $N_{T} = 0.4$, $N_{T} = 0.2$, $Sc = 0.3$, $K_{1} = 0.6$ and $Pr = 1.5$. The variation of $K_{1}$ and the magnitude of $\theta^{\prime } \left( 0 \right)$ and $\phi^{\prime } \left( 0 \right)$ presented in Table 2. The magnitude of $\theta^{\prime } \left( 0 \right)$ reduced and magnitude of $\phi^{\prime } \left( 0 \right)$ increased by improving values of $K_{1}$ with fix values of $D_{f} = 0.2$, $S_{r} = 0.3$, $Rd = 0.3$, $N_{b} = 0.2$, $N_{T} = 0.4$, $K = 0.3$, $Sc = 0.3$ and $Pr = 1.5$. The variation of $Pr$ and the magnitude of $\theta^{\prime } \left( 0 \right)$ and $\phi^{\prime } \left( 0 \right)$ presented in Table 2. The magnitude of $\theta^{\prime } \left( 0 \right)$ increased and magnitude of $\phi^{\prime } \left( 0 \right)$ reduced by improving values of $Pr$ with fix values of $D_{f} = 0.2$, $S_{r} = 0.3$, $Rd = 0.3$, $N_{b} = 0.2$, $N_{T} = 0.4$, $K = 0.3$, $Sc = 0.3$ and $K_{1} = 0.6$. The comparison of our results with Chakraborty et al.^67^ are presented in Table 3 for different values of $Pr.$ It is good agreement with Chakraborty et al.^67^.

**Table 2:** Numerical results of $- \theta^{\prime } \left( 0 \right)$ and $- \phi^{\prime } \left( 0 \right)$ for different values of physical parameters.

$D_{f}$	$S_{r}$	$Rd$	$N_{B}$	$N_{T}$	$Sc$	$K$	$K_{1}$	$Pr$	$- \theta^{\prime } \left( 0 \right)$	$- \phi^{\prime } \left( 0 \right)$
0.1	0.3	0.3	0.2	0.4	0.7	0.3	0.6	1.5	0.4886	2.0579
0.2	–	–	–	–	–	–	–	–	0.4187	2.0666
0.3	–	–	–	–	–	–	–	–	0.3468	2.0735
0.4	–	–	–	–	–	–	–	–	0.2728	2.0789
0.2	0.0	–	–	–	–	–	–	–	0.3466	2.0487
–	0.3	–	–	–	–	–	–	–	0.3468	2.0735
–	0.6	–	–	–	–	–	–	–	0.3470	2.0979
–	0.9	–	–	–	–	–	–	–	0.3473	2.1216
–	0.3	0.0	–	–	–	–	–	–	0.3854	2.0688
–	–	0.3	–	–	–	–	–	–	0.3468	2.0735
–	–	0.6	–	–	–	–	–	–	0.3150	2.0772
–	–	0.9	–	–	–	–	–	–	0.2889	2.0803
–	–	0.3	0.1	–	–	–	–	–	0.3543	2.0727
–	–	–	0.2	–	–	–	–	–	0.3468	2.0735
–	–	–	0.3	–	–	–	–	–	0.3395	2.0744
–	–	–	0.4	–	–	–	–	–	0.3325	2.0754
–	–	–	0.2	0.1	–	–	–	–	0.4467	2.0723
–	–	–	–	0.2	–	–	–	–	0.4113	2.0728
–	–	–	–	0.3	–	–	–	–	0.3780	2.0730
–	–	–	–	0.4	–	–	–	–	0.3468	2.0735
–	–	–	–	0.4	0.1	–	–	–	0.6317	0.6511
–	–	–	–	–	0.3	–	–	–	0.5131	1.2411
–	–	–	–	–	0.5	–	–	–	0.4226	1.6957
–	–	–	–	–	0.7	–	–	–	0.3468	2.0735
–	–	–	–	–	0.7	0.0	–	–	0.4553	2.1033
–	–	–	–	–	–	0.3	–	–	0.3468	2.0735
–	–	–	–	–	–	0.6	–	–	0.2796	2.0548
–	–	–	–	–	–	0.9	–	–	0.2341	2.0421
–	–	–	–	–	–	0.3	0.0	–	0.5571	0.8450
–	–	–	–	–	–	–	0.3	–	0.4949	1.1840
–	–	–	–	–	–	–	0.6	–	0.3468	2.0735
–	–	–	–	–	–	–	0.9	–	− 0.2700	9.2854
–	–	–	–	–	–	–	0.6	1.0	0.3208	2.0946
–	–	–	–	–	–	–	–	1.5	0.3417	2.0781
–	–	–	–	–	–	–	–	2.0	0.3523	2.0679
–	–	–	–	–	–	–	–	2.5	0.3580	2.0609

**Table 3:** Comparative results of Sherwood and Nusselt number for different values of $Pr$ and rest of physical values dimension.

$Pr$	Chakraborty et al.^67^		Present analysis	
	Sherwood Number	Nusselt Number	Sherwood Number	Nusselt Number
0.7	$0.34689118$ 0	$1.61983352$ 0	$0.3468912$ 0	$1.619336$ 0
1.00	$0.57428288$ 0	$1.80285833$ 0	$0.5742829$ 0	$1.802859$ 0
3.00	$1.15942580$ 0	$2.33075212$ 0	$1.1594260$ 0	$2.330753$ 0
5.00	$1.56331503$ 0	$2.71619820$ 0	$1.5633150$ 0	$2.716199$ 0

## Final remarks

Maxwell fluid at vertical exponential stretching sheet under second order slip effect is deliberated. Dufour and Soret impact for vertical exponential stretching sheet under nonlinear radiation are deliberated. Thermal and concentration slips with viscous dissipation are taken into account under the Buongiorno’s model. Under the above assumptions, the differential model constructed using the boundary layer approximations using the governing equations. The similarities transformations are introduced which applied the differential model (partial differential equations) and developed the dimensionless differential equations (ordinary differential equations). The dimensionless differential equations are cracked by numerical scheme. The main results of the physical parameters are presented as below:

- The velocity of fluid declined by boosting values of $M$. In fact, increasing magnetic field value increases the external magnetic field. This increase in the external magnetic field causes a wall parallel Lorentz force, which slows the expansion of the momentum boundary layer. When looking at the figure closely, it can be seen that the velocity suddenly declined towards the plate as the magnetic field increases.
- The curves of fluid velocity enhanced due to increasing the values of $\lambda$. Velocity slip is a fluid-boundary interaction in physics. If the velocity slip increased, the fluid velocity profile would eventually become increasing.
- Temperature curves declined by improving values of $K_{1}$. The thermal thickness reduced when improved the values of $K_{1}$.
- Values of $Rd$ and fluid temperature found to be same behavior of increasing found. Physically, radiation increasing means energy increased as well as temperature of fluid increased.
- Temperature function increased due to improving values of $N_{T}$. As the values of $N_{T}$ increased which enhanced the temperature due to $N_{T}$ has high gradient temperature.
- The $D_{f}$ parameter increased which declined the curves of fluid concentration. Physically, $D_{f}$ (diffusion-thermo) values enhanced which reduced the concentration function.

## Data Availability

The datasets generated and/or analyzed during the current study are not publicly available but are available from the corresponding author on reasonable request.

## List of symbols

${D}_{B}({m}^{2}/s)$: Brownian diffusion coefficient
$u,v (m/s)$: Velocity along *x*-and *y*-direction
$$C$$: Concentration
$$T(K)$$: Temperature
$${C}_{\infty }$$: Ambient concentration
$${T}_{\infty }(K)$$: Ambient temperature
$${C}_{w}$$: Wall concentration
$${T}_{w}(K)$$: Wall temperature
$$g(\mathrm{m}/{s}^{2})$$: Gravity
${D}_{T}({m}^{2}/\mathrm{s})$: Thermal diffusivity coefficient
${q}_{w}(kg/{m}^{2}s)$: Surface mass flux
$$ \mu (kg/ms)$$: Dynamic viscosity
$$\nu ({m}^{2}/s)$$: Kinematic viscosity
${\rho }_{f}(kg/{m}^{3})$: Density of fluid
$\beta$: Maxwell fluid parameter
$$M$$: Magnetic field
$${\lambda }_{1}$$: Second orderslip
$$K$$: Thermal slip
$$Rd$$: Radiation parameter
$$k (W/mK)$$: Thermal conductivity
$$S{h}_{x}$$: Sherwood number
$${N}_{B}$$: Brownian motion
$$N{u}_{x}$$: Nusselt number
$$R{e}_{x}$$: Reynolds number
$${N}_{T}$$: Thermophoresis parameter
$$\mathrm{Pr}(\nu /\alpha )$$: Prandtl number
$$Sc(\nu /{D}_{B})$$: Schmidt number
${D}_{B}({m}^{2}/\mathrm{s})$: Mass diffusivity coefficient
${c}_{p}(J/kgK)$: Specific heat capacity
${B}_{0}$: External magnetic field
$$f(\eta )$$: Dimensionless velocity
$$\theta (\eta )$$: Dimensionlesstemperature
$${D}_{f}$$: Dufour number
$${S}_{r}$$: Soret number
$$\lambda $$: Velocity slip
$${N}_{c}$$: Bouncyforce
$${K}_{1}$$: Concentration slip
${D}_{T}({m}^{2}/\mathrm{s})$: Thermal diffusivity coefficient
